# Bilateral Vestibular Dysfunction Associated With Chronic Exposure to Military Jet Propellant Type-Eight Jet Fuel

**DOI:** 10.3389/fneur.2018.00351

**Published:** 2018-05-16

**Authors:** Terry D. Fife, Michael J. A. Robb, Kristen K. Steenerson, Kamala C. Saha

**Affiliations:** ^1^Barrow Neurological Institute, Phoenix, AZ, United States; ^2^Robb Oto-Neurology Clinic, Phoenix, AZ, United States; ^3^Stanford University, Palo Alto, CA, United States

**Keywords:** JP-8, jet propulsion fuel-8, JP-8 jet fuel, bilateral vestibular dysfunction, ototoxicity, vestibulotoxicity, rotational chair, hydrocarbons

## Abstract

We describe three patients diagnosed with bilateral vestibular dysfunction associated with the jet propellant type-eight (JP-8) fuel exposure. Chronic exposure to aromatic and aliphatic hydrocarbons, which are the main constituents of JP-8 military aircraft jet fuel, occurred over 3–5 years’ duration while working on or near the flight line. Exposure to toxic hydrocarbons was substantiated by the presence of JP-8 metabolite *n*-hexane in the blood of one of the cases. The presenting symptoms were dizziness, headache, fatigue, and imbalance. Rotational chair testing confirmed bilateral vestibular dysfunction in all the three patients. Vestibular function improved over time once the exposure was removed. Bilateral vestibular dysfunction has been associated with hydrocarbon exposure in humans, but only recently has emphasis been placed specifically on the detrimental effects of JP-8 jet fuel and its numerous hydrocarbon constituents. Data are limited on the mechanism of JP-8-induced vestibular dysfunction or ototoxicity. Early recognition of JP-8 toxicity risk, cessation of exposure, and customized vestibular therapy offer the best chance for improved balance. Bilateral vestibular impairment is under-recognized in those chronically exposed to all forms of jet fuel.

## Introduction

The primary jet fuel used in the United States Air Force and NATO military operations is jet propellant type-eight (JP-8). JP-8 is a kerosene-based fuel comprised of over 228 aromatic and aliphatic hydrocarbons (1). During 1992–1996, the Air Force transitioned from using JP-4 to JP-8 due to the improved safety profile of the latter. JP-8 is also used as a multipurpose fuel for ground vehicles, generators, tent heaters and air conditioners, lamps, and cooking stoves allowing for an array of exposure opportunities. JP-8 typically contains 18% aromatic hydrocarbons and 82% aliphatic hydrocarbons, in particular, 9% C8–C9, 65% C10–C14, and 7% C15–C17 (2). JP-8 differs from commercial airline fuel due to its military additives including static electricity/corrosion/icing inhibitors, thermal stability enhancers, and antioxidants.

Vestibulotoxicity from JP-8 has been suggested but not well-documented in previous studies. Several studies indicate an association with impaired balance (3, 4), hearing, and central auditory processing (5–8).

We present a case study of three patients who had chronic complaints of dizziness, headache, fatigue, and imbalance. One patient performed fuel-tank maintenance for the Air National Guard for over a decade, while the other two worked 4–6 years in administrative positions in a small under-ventilated building proximate to the flight line. Each developed documented-bilateral vestibular dysfunction most probably related to chronic inhaled JP-8 fumes over a long period of time.

## Case Reports

### Case 1: Military Flight Refueler

A 37-year-old woman presented with several years of progressively worsening continuous dizziness, headache, and fatigue. The dizziness consisted of sensations of spinning, tilting, disequilibrium, and head fullness. She did not report tinnitus or hearing loss. She was employed as a military flight refueler and exposed to JP-8 vapors and exhaust while working full-time on and around a KC-135E tanker aircraft, a plane used for performing in-flight refueling missions. She worked in a large enclosed hangar that housed all but the tail section of the tanker aircraft. During inspection and maintenance of the aircraft, up to 9,750 gallons of fuel would be loaded. Jet fuel vapors were always present in the hangar due to venting, small leaks, and fuel residue. Fuel vapor concentrations were even greater when engine maintenance necessitated removal of fuel filters and fuel components, draining of fuel into buckets, and opening of fuel lines. She worked in engine maintenance with over 4 years of inhalational and dermal exposure to JP-4 and JP-8.

Her examination showed moderately impaired equilibrium to walk only three steps in tandem before taking a sidestep. Romberg testing revealed more sway during eye closure but no falling. Her medical and neurological examinations were normal. There was no spontaneous, gaze, or positional nystagmus. Qualitative head impulse test was not performed at that time.

**Figure 1:**
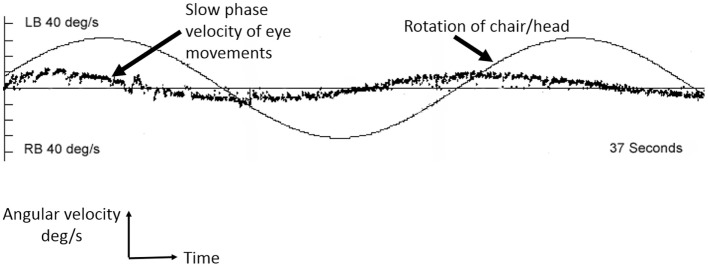
Example of vestibular hypofunction seen across all frequencies for Case 1. A sinusoidal rotation at 0.04 Hz, 60 deg/s by rotational chair testing. The smooth sinusoid is the chair/head rotation at 0.04 Hz, and the scatter line represents the slow phases of compensatory nystagmus in response to the sinusoidal head (and chair) rotations. The abscissa is time measured in seconds. LB, left beating nystagmus; RB, right beating nystagmus. Performed using Micromedical Technologies (Chatham, IL, USA).

**Figure 2:**
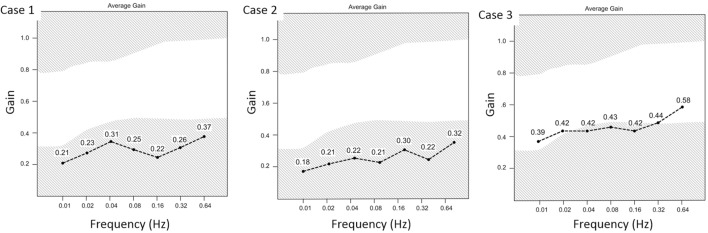
The summary of average gain values at frequencies between 0.01 and 0.64 Hz sinusoidal rotations. The gain values were below normal for all frequencies tested for Cases 1 and 2 and were reduced at most frequencies for Case 3.

A brain SPECT study at an outside facility revealed mild-right frontal hypoperfusion that persisted on a repeated study the following year. Neurocognitive examination showed overall memory function in the 97th percentile. An MRI brain without gadolinium and an EEG were normal. An initial hydrocarbon assay revealed the presence of 3-methylpentane and *n*-hexane in the blood at concentrations of 27 and 15.7 ng/ml (parts per billion), respectively (none should be measurable in normal individuals). Ten months later, 3-methylpentane and *n*-hexane remained present although at significantly lower concentrations. Eighteen months after presentation, 3-methylpentane and *n*-hexane persisted in the blood and had only diminished an additional 20%. Rotational chair, more so than caloric vestibular testing, demonstrated bilateral vestibular dysfunction (Figures 1 and 2) with reduced gain values on step velocity along with a reduced time constant. Gain was also reduced on all sinusoidal rotations with increase phase lead at 0.01, 0.02, and 0.04 Hz rotations. The patient reported that her headaches, dizziness, fatigue, and mild unsteadiness improved somewhat following a transfer to the finance department where no JP-8 exposure existed. There was a long interval of 15 years since her initial visit when she was lost to follow up. Now, 16 years after her initial presentation, she reports that the dizziness is mild but headaches and severe fatigue persist. She has continued to work but plans to retire earlier than originally anticipated due to the ongoing symptoms. Recently, cervical and ocular vestibular-evoked myogenic potential (cVEMP and oVEMP, respectively) and video head impulst testing for each canal were performed and all results were normal.

### Cases 2 and 3

The following two patients were employees in a small-purchasing warehouse, located 75 feet south of the flight path, which was separated from the blast and heat emissions from jet aircraft engines by a metal-coated and chain-link fence. Neither air conditioning vents nor carpet had not been cleaned or replaced for over a decade. On inspection, the vents were found to be malfunctioning such that air was able to enter the building but unable to escape. Subsequent inspection by the U. S. Occupational Safety and Health Administration (OSHA) confirmed poor ventilation evidenced by carbon dioxide concentrations >1,500 ppm (normal <1,000 ppm according to the U.S. Department of Labor). Hydrocarbons discovered in the carpet *via* an independent analysis using gas chromatography/mass spectrometry included undecane (C11), dodecane (C12), tridecane (C13), tetradecane (C14), and toluene (C8)—all known JP-8 constituents (2). The chemicals present in the office carpet likely reflected poor indoor air quality. Vapor, aerosol, dermal, and eye absorption of JP-8 are presumed.

### Case 2: Warehouse Employe 1

A 45-year-old female contracting officer for the National Guard reported several years of imbalance, headache, fatigue, eye and skin irritation, coughing, sinus congestion, recurrent urinary tract infections, chest tightness, irritability, depression, shortness of breath, palpitations, and numbness. She described her dizziness as an intermittent floating and a rightward tilting sensation with imbalance lasting minutes to hours without any particular pattern. She had a history of asthma and allergies including reaction to aspirin causing urticaria and airway obstruction. In 1998, she developed syncope and dizziness though no specific cause was found. She started working in the building in 1994 and worked there full-time for 5 years.

Her examination was normal except that she fell on Romberg testing and could only walk a few steps in tandem. Brain MRI, EEG, audiogram, and pulmonary function tests were normal. Quantitative rotational chair and caloric vestibular tests revealed bilateral peripheral vestibular dysfunction (Table 1; Figure 2). Her caloric responses improved following removal from the under-ventilated environment (Table 1).

**Table 1:** Caloric vestibular test results of each case.

	Timing^b^	RC^a^	RW^a^	LC^a^	LW^a^	VR%	DP%
Case 1	0	16	12	14	20	9 Right	16 Left
Case 2	0	5	7	6	8	7 Right	0
Case 2	16 Months	19	32	30	21	0	22 Right
Case 3	0	23	17	11	10	31 Left	8 Right
Case 3	7 Months	20	32	20	36	3 Right	3 Left

*All studies performed with water caloric irrigation using ICS Chartr VNG, now GN Otometric (Schaumburg, IL)*.

*^a^Peak slow phase velocity in degrees/second of caloric-induced nystagmus*.

*^b^Time 0 = initial presentation. Subsequent studies designated as months subsequent to presentation*.

*RC, right cool irrigation, LC, left cool irrigation, RW, right warm irrigation, LW, left warm irrigation; VR, vestibular response asymmetry as a percentage using Jongkees formula; DP, directional preponderance which reflects the direction (rightward versus leftward) of the bithermal caloric-induced nystagmus expressed as a percentage using Jongkees formula*.

### Case 3: Warehouse Employe 2

A 54-year-old female National Guard contract specialist presented with 2 years of intermittent dizziness, blurred vision, and occasional palpitations. Dizziness was experienced at least 3 days a week. She reported intermittent problems with erratic heart beats, cough, sneezing, headaches, fatigue, recurrent sinus infections, upper respiratory tract, and bladder infections. She worked in the purchasing warehouse full-time for 3 years. When away from the workplace her symptoms were improved. After moving with her colleagues into a new building, the frequency of dizziness was lessened.

Her medical, neurological, and oto-neurological examinations were normal. Electronystagmography revealed somewhat reduced caloric vestibular responses for age and a 33% reduced vestibular response on the left. Rotational testing showed reduced gain with sinusoidal rotational stimuli at frequencies from 0.02 to 0.16 Hz (Figure 2). Her caloric responses improved following removal from the “sick-building” environment (Table 1). Computerized dynamic posturography showed falls on conditions 4, 5 and 6 indicating some general impairment of equilibrium and a predominant vestibular deficit pattern. Audiometric tests were normal except for mild-sensorineural hearing loss in the right ear from 250 to 8,000 Hz and borderline normal left-sided hearing from 250 to 2,000 Hz sloping to a moderate loss between 3,000 and 8,000 Hz.

## Discussion

These case reports describe three women working in close proximity to JP-8 jet fuel who developed bilateral vestibulopathy after 3–5 years of exposure. Serum studies in one of the patients (Case 1) demonstrated JP-8 fuel metabolites 3-methylpentane and *n*-hexane (1). These compounds are not present in human blood normally. The levels of these metabolites diminish over time once the individual is removed from repeated exposure. Quantitative vestibular testing revealed bilateral vestibular dysfunction in all three patients after JP-8 exposure. There was no other probable identifiable explanation for the vestibular dysfunction. Although causal relationship cannot be definitively proven yet, this collection of data suggests a relationship between prolonged exposure to JP-8 fuel and development of bilateral vestibular dysfunction which has not previously been documented in humans.

The presence of bilateral vestibular dysfunction in these cases may be due to a process localizing to the vestibular nerves, the vestibular end-organs, or a combination of both. However, the constituent hydrocarbons in JP-8 are lipophilic and have been shown to affect the CNS so a peripheral vestibular mechanism is assumed but not assured. Indeed, for Case 1 on whom we have long-term follow up, headaches and severe fatigue have persisted for years, which are symptoms associated with CNS hydrocarbon toxicity. A CNS toxicity contribution might also explain the chronicity of symptoms and lackluster response to vestibular rehabilitative efforts. The relative preservation of caloric vestibular responses (Table 1) in the presence of prominent pan-frequency vestibular dysfunction on rotational chair testing raises the possibility of some degree of frequency-specific ototoxicity (9). Furthermore, improvement in the caloric responses with removal from continued exposure implies the possibility of some degree of reversibility of vestibular dysfunction. Dedicated occupational studies in humans on the vestibular effects of chronic JP-8 exposure are limited and data are still sparse on the direct mechanisms of ototoxicity due to jet fuel.

### Human Studies

A study of the effects of low-level exposure to JP-8 fuel vapor in U.S. Air Force aircraft maintenance personnel found a correlation between solvent exposure (benzene, toluene, xylene) and increased postural sway implying vestibular or proprioceptive impairment (3). Another study of 37 Air Force personnel with short-term work day exposure to JP-8 did not identify increases in postural sway (4). Long-term exposure to jet fuel in a subset of eight subjects assessed by vestibular testing found minor vestibular abnormalities but those patients actually reported more cognitive symptoms than vestibular findings (10).

Liquid hydrocarbon fractions are distilled from petroleum based on density. Although there may be variations in composition, these hydrocarbon mixtures have toxic effects on the human body similar to jet fuels (11). Some organic solvents commonly used in commercial industries are also hydrocarbon mixtures and would be expected to have similar toxicities. Indeed, dizziness, sometimes but not always resulting in vestibular test abnormalities, is a common symptom among individuals exposed to organic solvents (12). Workers exposed chronically to toluene and ethanol for many years exhibited reduced pursuit tracking and increased postural sway; and the latter suggests possible impairment of vestibular function (13). A study of three welders with short-term exposure to hydrocarbons found vestibular nystagmus and vestibular abnormalities that persisted for 3–18+ months after exposure (14).

It has been suggested that aliphatic and aromatic hydrocarbon toxicity may be associated with bilateral vestibular dysfunction, dizziness, and abnormal performance on posturography testing (3, 15). Although, organic solvents may have toxic effects on peripheral vestibular function or brainstem vestibular pathways (16), most of the data simply suggest increased sway in those exposed, which is not necessarily a specific indicator of vestibular dysfunction. A small study of 18 individuals with exposure to organic solvents found a significantly greater number with abnormal vestibular function including oVEMP and cVEMP, and caloric testing when compared to unexposed controls. The authors suggest that organic solvent toxicity may adversely affect the function of the utricle and saccule to a greater degree than hearing or semicircular canal function (17).

### Animal Studies

Studies in rats exposed to JP-8 vapor for 6 h per day, 5 days per week for a total of 1 month showed that pure-tone hearing thresholds, outer hair cell function, and hair cell numbers remained unaffected with exposure of 1,500 mg/m^3^. However, when rats were exposed to JP-8 plus noise, marked decreases in distortion produce otoacoustic emissions amplitude, increases in pure-tone auditory threshold along with a small reduction (<1%) in the number of cochlear outer hair cells were detected (18, 19). A study of 26 pigmented rats exposed to toluene in a prosptective cross-over control study found a dose-related reduction in VOR suppression and reduced VOR gain and time constants (20). Another study in rats exposed to 1,000 mg/m^3^ of JP-8 found impaired encoding of stimulus intensity both in rats exposed only to JP-8 and in those exposed to JP-8 and noise. There were no changes in auditory thresholds and no loss of cochlear outer hair cells; however, there was impaired brainstem encoding of stimulus intensity indicating dysfunction of central auditory processing (6, 8).

There are no studies of the long-term effects of JP-8 specifically on peripheral vestibular function in humans. This may be in part because many exposed personnel tolerate limited exposure well, and those that do have symptoms have not been evaluated and reported in published literature. Bilateral vestibular dysfunction, regardless of cause, is probably under-recognized in clinical medicine (21). Hence, the true incidence of vestibulopathy from jet fuel exposure is unknown.

### Human Exposure and Absorption of Jet Fuel

Military duties such as fuel transportation, aircraft fueling and defueling, aircraft maintenance, cold aircraft engine starts, maintenance of equipment and machinery, use of tent heaters, and cleaning or degreasing with fuel may result in jet fuel exposure. Fuel handlers, mechanics, flight line personnel, especially crew chiefs, and even incidental workers remain at risk for developing illness secondary to chronic JP-8 fuel exposure in aerosol, vapor or liquid form. JP-8 is one of the most common occupational chemical exposures in the US military (1). The Air Force has set recommended exposure limits for JP-8 at 63 ppm (447 mg/m^3^ as an 8-h time-weighted average) (22).

In addition to exposure by JP-8 vapor inhalation, toxicity may also occur by absorption through the skin, which is proportional to the amount of skin exposed and the duration of exposure (23, 24). In addition to the standard operating procedure and safety guidelines, double gloving, immediate onsite laundering of contaminated/soiled jumpsuits, regular washing of safety goggles and masks, reduced foam handling time, smoking cessation, adequate cross ventilation, and frequent shift breaks may reduce the overall risk of JP-8 induced illness (1, 2). At this time, OSHA has not determined a legal limit for jet fuels in workroom air. The U.S. National Institute of Occupational Safety and Health set a recommended limit of 100 mg/m^3^ for kerosene in air averaged over a 10-h work day. Multi-organ toxicity has been documented from JP-8 exposure in animal experiments over the past 15 years. More recently, toxicology researchers are investigating the adverse tissue effects of JP-8 jet fuel in concentrations *well below* permissible exposure limits. Ultimately, the new data may help us to better understand the emerging genetic, metabolic and inflammatory mechanisms underpinning JP-8 cellular toxicity—including auditory and vestibular toxicity—and lead to a reassessment of the safe JP-8 exposure limits (25, 26). In the meantime, bedside vestibular screening for vestibular dysfunction can be performed by dynamic visual acuity testing or by head impulse testing.

Are there any known JP-8 biomarkers? Yes. Breath, blood, urine, and microRNA tissue biomarkers have been studied and aid in confirming JP-8 exposure. Self-reported JP-8 exposure in the workplace is a reliable indicator and a stronger predictor of measured exposure than job title (27). After controlling for work shift smoking, measurements of blood volatile organic compounds (ethylbenzene, toluene, xylene) are higher among US Air Force personnel self-reporting JP-8 exposure in association with elevated hydrocarbons in the breathing zone (28). Urinary biomarkers 1- and 2-naphthol, the metabolites of naphthalene, are the most sensitive and useful short-term surrogates of JP-8 exposure due to their strong correlation with breathing zone naphthalene, greater abundance, and slower elimination kinetics (29, 30). Blood microRNAs (miRNAs) may be unique biomarkers for volatile organic compounds and have been compared recently to urinary biomarkers in human dockyard workers found to have toluene, xylene, and ethylbenzene in whole blood. Fifty subjects underwent miRNA microarray analysis and 211–695 mRNAs were identified for toluene, xylene, and ethylbenzene suggesting higher sensitivity, specificity, and accuracy than urinary biomarkers (31). The analysis of circulating miRNAs in the blood of military veterans exposed to JP-8 is worthy of future research.

## Conclusion

Bilateral vestibular dysfunction in these three patients with prolonged vapor and dermal JP-8 fuel exposure should raise awareness in people with occupations that expose them to jet fuels, liquid hydrocarbons, or organic solvents. Dizziness and mild imbalance may be the main initial symptoms. Early recognition and limiting further exposure as well as treatment with vestibular therapy (32) may improve their function and quality of life.

## Ethics Statement

Written informed consent to publish the report was obtained from each patient. This report was approved by the local Institutional Review Board at Barrow Neurological Institute/DignityHealth, Inc., case series tracking number Case Series 18-004.

## Author Contributions

TF attended to the three patients in oto-neurological consultation, contributed to project conception, data collection and analysis, critical revision, and final approval of the manuscript. MR contributed to project conception, scientific poster presentation, data collection and analysis, drafting of the article and critical revision, and final approval of the manuscript. KriS contributed to drafting of the article and critical revision and final approval of the manuscript. KaS contributed to critical revision and final approval of the manuscript.

## Conflict of Interest Statement

The authors declare that the research was conducted in the absence of any commercial or financial relationships that could be construed as a potential conflict of interest.

## References

[1] Ritchie GD Still KR Rossi J III Bekkedal MYV Bobb AJ Arfsten DP. Biological and health effects of exposure to kerosene-based jet fuels and performance additives. J Toxicol Environ Health B Crit Rev (2003) 6:357–451. 10.1080/10937400306473 12775519

[2] McDougal JN Pollard DL Weisman W Garrett CM Miller TE. Assessment of skin absorption and penetration of JP-8 jet fuel and its components. Toxicol Sci (2000) 55:247–55. 10.1093/toxsci/55.2.247 10828255

[3] Smith LB Bhattacharya A LeMasters G Succop P Puhala E II Medvedovic M Effect of chronic low-level exposure to jet fuel on postural balance of US Air Force personnel. J Occup Environ Med (1997) 39:623–32. 10.1097/00043764-199707000-00007 9253723

[4] Maule AL Heaton KJ Rodrigues E Smith KW McClean MD Proctor SP. Postural sway and exposure to jet propulsion fuel 8 among US Air Force personnel. J Occup Environ Med (2013) 55(4):446–53. 10.1097/JOM.0b013e31827db94b 23532195

[5] Fechter LD Gearhart CA Fulton S. Ototoxic potential of JP-8 and a Fischer-Tropsch synthetic jet fuel following subacute inhalation exposure in rats. Toxicol Sci (2010) 116:239–48. 10.1093/toxsci/kfq110 20378580

[6] Guthrie OW Wong BA McInturf SM Reboulet JE Ortiz PA Mattie DR. Inhalation of hydrocarbon jet fuel suppress central auditory nervous system function. J Toxicol Environ Health (2015) 78:1154–69. 10.1080/15287394.2015.1070389 26408153

[7] Guthrie OW Wong BA McInturf SM Reboulet JE Ortiz PA Mattie DR. Background noise contributes to organic solvent induced brain dysfunction. Neural Plast (2016) 2016:8742725. 10.1155/2016/8742725 26885406 PMC4739468

[8] Warner R Fuente A Hickson L. Jet fuel, noise, and the central auditory nervous system: a literature review. Mil Med (2015) 180:950–5. 10.7205/MILMED-D-14-00733 26327546

[9] Prepageran N Kisilevsky V Tomlinson D Ranalli P Rutka J. Symptomatic high frequency/acceleration vestibular loss: consideration of a new clinical syndrome of vestibular dysfunction. Acta Otolaryngol (2005) 125:48–54. 10.1080/00016480410017981 15799574

[10] Odkvist LM Arlinger SD Edling C Larsby B Bergholtz LM. Audiological and vestibulo-oculomotor findings in workers exposed to solvents and jet fuel. Scand Audiol (1987) 16:75–81. 10.3109/14992028709042159 3498206

[11] Kamal A Malik RN Fatima N Rashid A. Chemical exposure in occupational settings and related health risks: a neglected area of research in Pakistan. Environ Toxicol Pharmacol (2012) 34:46–58. 10.1016/j.etap.2012.02.009 22445870

[12] Gyntelberg F Vesterhauge S Fog P Isager H Zilstorff K. Acquired intolerance to organic solvents and results of vestibular testing. Am J Ind Med (1986) 9:363–70. 10.1002/ajim.4700090408 3706310

[13] Herpin G Gauchard GC Vouriot A Hannhart B Barot A Mur JM Impaired neuromotor functions in hospital laboratory workers exposed to low levels of organic solvents. Neurotox Res (2008) 13:185–96. 10.1007/BF03033502 18522898

[14] Hodgson WJ Furman J Ryan C Durrant J Kern E. Encephalopathy and vestibulopathy following short-term hydrocarbon exposure. J Occup Med (1989) 31:51–4. 2738752

[15] Hodgkinson L Prasher D. Effects of industrial solvents on hearing and balance: a review. Noise Health (2006) 8:114–33. 10.4103/1463-1741.33952 17704602

[16] Zamysłowska-Szmytke E Sliwińska-Kowalska M The influence of organic solvents on hearing and balance: a literature review. Med Pr (2013) 64:83–102. 10.13075/mp.5893/2013/0009 23650771

[17] Hsu PC Cheng PW Young YH. Ototoxicity from organic solvents assessed by an inner ear test battery. J Vestib Res (2015) 25:177–83. 10.3233/VES-150559 26756133

[18] Fechter LD Gearhart C Fulton S Campbell J Fisher J Na K JP-8 jet fuel can promote auditory impairment resulting from subsequent noise exposure in rats. Toxicol Sci (2007) 98:510–25. 10.1093/toxsci/kfm101 17483120

[19] Fechter LD Fisher JW Chapman GD Mokashi VP Ortiz P Reboulet JE Subchronic JP-8 jet fuel exposure enhances vulnerability to noise-induced hearing loss in rats. J Toxicol Environ Health A (2012) 75:299–317. 10.1080/15287394.2012.652060 22409492

[20] Niklasson M Tham R Larsby B Eriksson B. Effects of toluene, styrene, trichloroethylene, and trichloroethane on the vestibulo-and opto-oculo motor system in rats. Neurotoxicol Teratol (1993) 15:327–34. 10.1016/0892-0362(93)90034-L 8277926

[21] van de Berg R van Tilburg M Kingma H. Bilateral vestibular hypofunction: challenges in establishing the diagnosis in adults. ORL J Otorhinolaryngol Relat Spec (2015) 77:197–218. 10.1159/000433549 26366566

[22] Dudley AC Peden-Adams MM EuDaly J Pollenz RS Kell DE. An aryl hydrocarbon receptor independent mechanism of JP-8 jet fuel immunotoxicity in Ah-responsive and Ah-nonresponsive mice. Toxicol Sci (2001) 59:251–9. 10.1093/toxsci/59.2.251 11158718

[23] Mattorano DA Kupper LL Nylander-French LA. Estimating dermal exposure to jet fuel (naphthalene) using adhesive tape strip samples. Ann Occup Hyg (2004) 48:139–46. 10.1093/annhyg/meh003 14990435

[24] Kim D Andersen ME Nylander-French LA. Dermal absorption and penetration of jet fuel components in humans. Toxicol Lett (2006) 165:11–21. 10.1016/j.toxlet.2006.01.009 16497449

[25] Guthrie OW Xu H Wong BA McInturf SM Reboulet JE Ortiz PA Exposure to low levels of jet-propulsion fuel impairs brainstem encoding of stimulus intensity. J Toxicol Environ Health A (2014) 77:261–80. 10.1080/15287394.2013.862892 24588226

[26] Wong SS Vargas J Thomas A Fastje C McLaughlin M Camponovo R In vivo comparison of epithelial responses for S-8 versus JP-8 jet fuels below permissible exposure limit. Toxicology (2008) 254(1–2):106–11. 10.1016/j.tox.2008.09.018 18930109 PMC2927360

[27] Merchant-Borna K Rodrigues EG Smith KW Proctor SP McClean MD. Characterization of inhalation exposure to jet fuel among U.S. Air Force personnel. Ann Occup Hyg (2012) 56(6):736–45. 10.1093/annhyg/mes014 22433121

[28] Maule AL Proctor SP Blount BC Chambers DM McClean MD. Volatile organic compounds in blood as biomarkers of exposure to JP-8 jet fuel among US Air Force personnel. J Occup Environ Med (2016) 58(1):24–9. 10.1097/JOM.0000000000000611 26716845 PMC7087458

[29] Serdar B Egeghy PP Waidyanatha S Gibson R Rappaport SM. Urinary biomarkers of exposure to jet fuel (JP-8). Environ Health Perspect (2003) 111(14):1760–4. 10.1289/ehp.6275 14594628 PMC1241720

[30] Smith KW Proctor SP Ozonoff AL McClean MD. Urinary biomarkers of occupational jet fuel exposure among Air Force personnel. J Expo Sci Environ Epidemiol (2012) 22(1):35–45. 10.1038/jes.2011.38 22044926

[31] Song MK Ryu JC. Blood miRNAs as sensitive and specific biological indicators of environmental and occupational exposure to volatile organic compound (VOC). Int J Hyg Environ Health (2015) 218(7):590–602. 10.1016/j.ijheh.2015.06.002 26141241

[32] Hall CD Herdman SJ Whitney SL Cass SP Clendandiel RA Fife TD Vestibular rehabilitation for peripheral vestibular hypofunction: an evidence-based clinical practice guideline: from the American Physical Therapy Association Neurology Section. J Neurol Phys Ther (2016) 40:124–55. 10.1097/NPT.0000000000000120 26913496 PMC4795094

